# Post-COVID-19 Myositis Based on Magnetic Resonance Imaging: A Case Report

**DOI:** 10.7759/cureus.30293

**Published:** 2022-10-14

**Authors:** Neha D Shetty, Rajasbala P Dhande, Vadlamudi Nagendra, Bhavik S Unadkat, Sheetal S Shelar

**Affiliations:** 1 Radiology, Jawaharlal Nehru Medical College, Datta Meghe Institute of Medical Sciences, Wardha, IND

**Keywords:** coronavirus, sars-cov-2, mri, myositis, covid 19

## Abstract

The severe acute respiratory syndrome Coronavirus 2 (SARS-CoV-2) causes COVID-19, which is known to cause fever, dry cough, exhaustion, headache, and loss of taste and smell. Although fever, sore throat, and cough have historically been the utmost characteristic symptoms of the illness, published case reports have recently started to emphasize additional uncommon and unusual presentations of infection with the coronavirus. In COVID, the musculoskeletal system is seldomly involved. In addition to reviewing the causes and imaging characteristics of COVID-19-related illnesses of the musculoskeletal system, we elaborate on a case of a middle-aged man who developed myositis as sequelae to the COVID-19 infection.

## Introduction

The coronaviruses (CoVs) are members of the order Nidovirales, family Coronaviridae, and genus Coronavirus. The official name of this virus is SARS-CoV-2. Seven CoV species are known to infect people. Only MERS-CoV and SARS- CoV have the capacity to lead to serious human illness. 

The remaining are linked to minor respiratory illnesses like the common cold. The average number of days that COVID takes to incubate is two to seven (range of one to two weeks). The single-stranded (positive-sense) RNA virus, SARS-CoV-2, which causes COVID-19, is known to produce fever, dry cough, fatigue, headache, and loss of taste and smell. Some COVID-19-affected individuals may develop a severe form of pneumonia, eventually leading to acute respiratory distress syndrome (ARDS) and respiratory failure. Recently published case reports have emphasized additional uncommon and unusual presentations of infection in individuals affected by the SARS-CoV-2 virus. SARS-CoV has the necessary potential to cause community and nosocomial transmission and result in severe morbidity and mortality. The virus also seems to involve the musculoskeletal system. Though these are believed to be infrequent COVID-19 symptoms, there are multiple case reports of myositis and rhabdomyolysis induced by COVID-19.

The illness also affects people globally on a moral or psychological, legal, and political level. These obstacles have a significant impact on the nation's economic progress. In this article, we discuss a case of a middle-aged man with myositis infected by COVID-19, as well as the pathophysiology and magnetic resonance imaging characteristics of the musculoskeletal system linked to COVID-19 [1].

## Case presentation

Presentation and examination

A 47-year-old male patient had a history of positive reverse transcription-polymerase chain reaction (RT-PCR) test for SARS-CoV2 infection three months before, for which he was hospitalized and consequently underwent treatment in the form of antibiotics, prophylactic thrombolytics, and supportive O2 therapy. At the time of presentation, he came to our tertiary care center in central India with complaints of pain and swelling over the right lower limb for 20 days. The pain was insidious in onset, gradual in progression, and was initially present over the posterior aspect of the right lower limb at the calf region and later progressed to the anterior compartment. The pain aggravated during physical activities and relieved on rest. The swelling was present on the right lower limb, which was smooth, diffuse in nature, and associated with redness and local rise of temperature on the overlying skin. There was no restriction to movements of the joint above and below the swelling, and no associated complaints of discharge from the swelling or visible pulsations were noted. The patient had no previous history of trauma, endocrine disorders, similar complaints in the family, blood disorders, or genetic disorders. The patient had no history of usage of lipid-lowering drugs, alcohol, colchicine, glucocorticoids, or antimalarial drugs in the past. The results of the neurological and muscle strength testing were within normal limits.

Laboratory investigations

The values of the complete blood count were as follows: Hemoglobin 14.5g/dL, Hematocrit 44%, RBC count 4.9×10^6^/microL, mean corpuscular volume (MCV) 89fL, mean corpuscular hemoglobin concentration (MCHC) 33, red blood cell distribution width (RDW) 12%, platelet count 230×10^3^/microL, WBC count 15,000/mcL. The WBC count was found to be raised. Biochemical tests for detection of inflammatory markers were performed, which revealed C-reactive protein (CRP) 30mg/L, erythrocyte sedimentation rate (ESR) 40mm/hr, lactate dehydrogenase (LDH) 748 IU/L and creatine kinase (CK) 644 IU/L. A significantly high value of CRP, ESR, lactate dehydrogenase (LDH), and CK was noted. Differential diagnoses of a nervous system disease such as encephalitis, myelitis, or Guillain-Barré syndrome were ruled out due to normal cognitive and cerebellar functions with normal reflexes. Tests for infections such as HIV, Hepatitis B and C viruses, and Lyme disease were negative. The patient had no history of travel to tropical areas or toxic drug exposure, thus ruling out these causes.

Imaging findings

**Figure 1:**
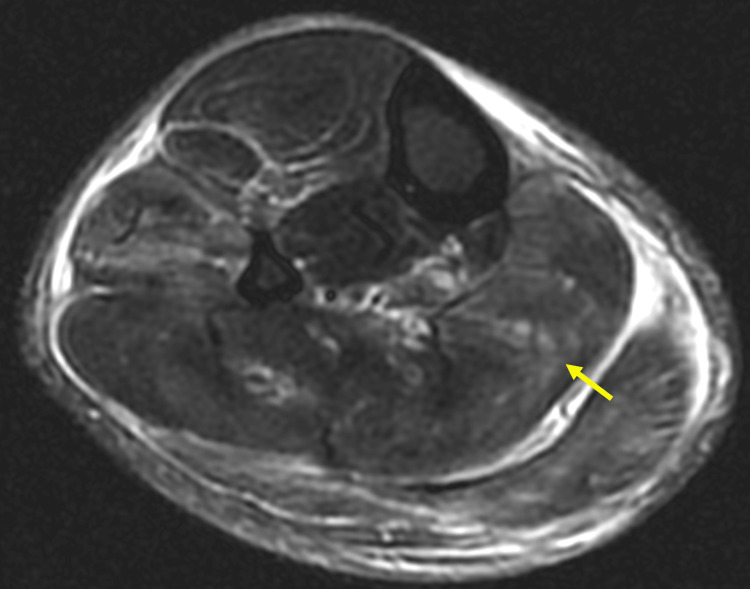
Axial section STIR sequence of right lower limb. Axial section STIR sequence of right lower limb showing extensive intramuscular hyperintensities in the anterior compartment (extensor digitorum longus), lateral (peroneus longus), and superficial posterior compartment (plantaris, gastrocnemius, and soleus) and intermuscular facial planes, predominantly in the proximal part. There are no e/o any necrotic changes.

**Figure 2:**
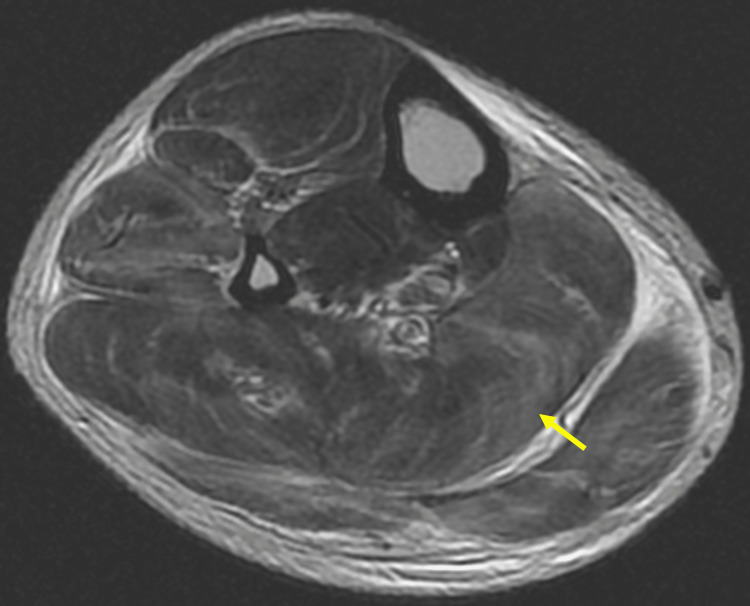
Axial section T2-weighted sequence of the right lower limb. Axial section T2-weighted sequence of right lower limb showing extensive intramuscular hyperintensities in the anterior compartment (extensor digitorum longus), lateral (peroneus longus), and superficial posterior compartment (plantaris, gastrocnemius, and soleus) and intermuscular facial planes, predominantly in the proximal part. There are no e/o necrotic changes.

**Figure 3:**
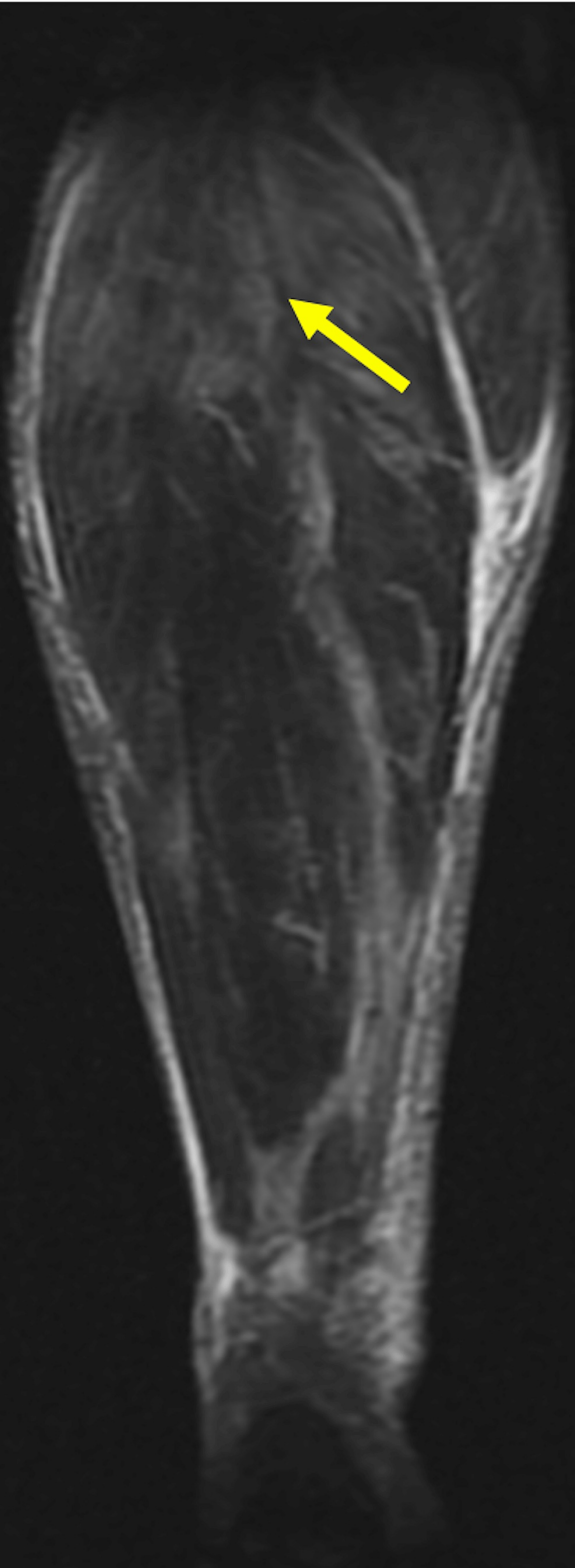
Coronal section STIR sequence of right lower limb Coronal section STIR sequence of right lower limb showing extensive intramuscular hyperintensities in the anterior compartment (extensor digitorum longus), lateral (peroneus longus), and superficial posterior compartment (plantaris, gastrocnemius, and soleus) and intermuscular facial planes, predominantly in the proximal part. There are no e/o necrotic changes.

**Figure 4:**
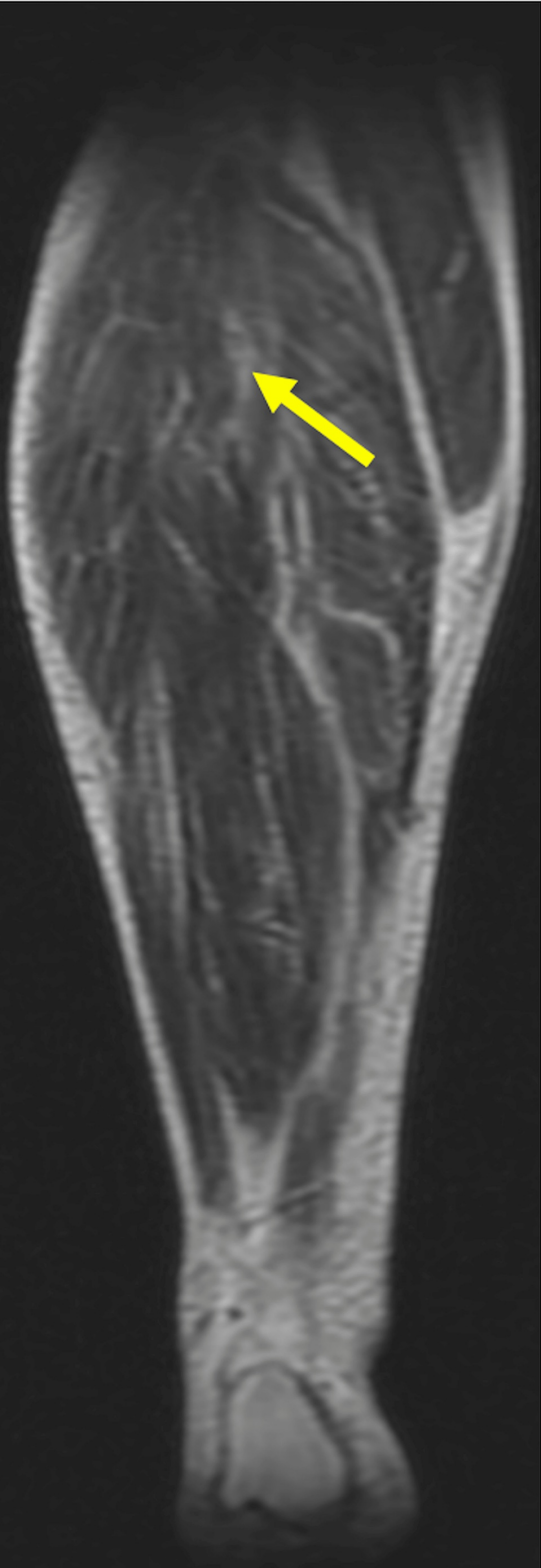
Coronal section T2-weighted sequence of the right lower limb. Coronal section T2-Weighted sequence of right lower limb showing extensive intramuscular hyperintensities in the anterior compartment (extensor digitorum longus), lateral (peroneus longus), and superficial posterior compartment (plantaris, gastrocnemius, and soleus) and intermuscular facial planes, predominantly in the proximal part. There are no e/o necrotic changes.

B-mode ultrasonography examination of the swelling showed increased diffuse homogenous echogenicity in the soleus, plantaris, and gastrocnemius muscles in the posterior compartment of the right lower limb. The extensor digitorum longus and peroneus longus were shown to exhibit enhanced echogenicity that was diffusely distributed along the muscles in the anterior compartment and lateral compartment, respectively. The patient was therefore suggested to undergo magnetic resonance imaging (MRI) for further evaluation. In the MRI of the right lower limb, there was evidence of extensive T2/STIR hyperintensities in muscles of the anterior compartment (extensor digitorum longus), lateral (peroneus longus), superficial posterior compartment (plantaris, gastrocnemius, and soleus) and intermuscular facial planes, predominantly in the proximal part (Figures 1-4). Inflammatory myositis secondary to COVID was considered the most probable diagnosis based on the MRI findings and associated positive history of COVID-19, three months ago.

Treatment and follow-up

A course of the nonsteroidal anti-inflammatory drug (NSAID), namely, Indomethacin 75mg OD, and immunomodulators such as glucocorticoids, namely, Prednisolone 50mg OD were advised to the patient for three weeks with tapering of Prednisolone dosage after two weeks. A month after receiving the therapy, the patient underwent a follow-up examination. The patient's symptoms had entirely subsided without a trace of the presenting complaints. The subsequent biochemical tests for inflammatory markers such as ESR, CK, and LDH readings had all returned to baseline.

## Discussion

The SARS-CoV-2 coronavirus was formally declared a pandemic on March 11, 2020 [2,3]. There have been over 21 million cases of COVID-19 as of August 2020, reported worldwide, with over 800 000 COVID-19-associated deaths [4]. The possibility of developing a severe form of the illness is more so in older age groups and patients with comorbidities such as cardiovascular disease, diabetes mellitus, and obesity. Corticosteroids, mechanical ventilation, thromboembolic prophylaxis, and oxygen therapy are potential therapeutic options for COVID-19 patients who are experiencing acute symptoms [5]. Treatment guidelines, prevention measures, and presentations of COVID-19 are currently being updated. The SARS-CoV-2 is a single-stranded (positive sense) RNA virus that is coupled with a nucleoprotein inside a capsid made of matrix protein in spherical or pleomorphic enclosed particles. In addition to the gastrointestinal system, urinary system (kidney and bladder), the pancreas, spleen, heart, and blood vessels, the Angiotensin-converting enzyme 2 (ACE2) receptor is highly expressed in lung epithelial cells [3,5].

Additionally, the central and peripheral neural systems, as well as skeletal muscle, contain ACE2 receptors. [6]. Angiotensin-converting enzyme 2 receptors are found in human cells and are recognized by this RNA virus. Viral release by cell apoptosis occurs after replication of the virus within human host cells [3,5]. Additionally, coronavirus induces an inflammatory response (including immunological responses, which are natural innate, and adaptive), which may lead to an overproduction of cytokines and eventually multi-organ destruction [3,5]. A patient affected with SARS-CoV2 may present without any debilitating symptoms or with mild to moderate upper respiratory tract infection (URTI) or with acute respiratory distress syndrome (ARDS). Initially, it was primarily known to affect the respiratory system. It is now recognized that SARS-CoV-2 infection can cause a wide range of extrapulmonary signs. Gastrointestinal symptoms, dysfunction of the heart, kidney, and liver, acute coronary syndrome, dermatologic abnormalities, and neurologic sequelae are the extrapulmonary manifestations noted. Although myalgia is a prevalent clinical symptom in SARS-CoV2 virus-affected individuals, early in the pandemic, additional musculoskeletal signs of COVID-19 were seldom reported [3-5]. Nevertheless, there have been more reports of rheumatologic problems and neuromuscular symptoms linked to the COVID-19 virus, the associated therapy, and course in the hospital as the number of patients and survivors increased globally [6].

COVID-19 musculoskeletal symptoms need to be carefully evaluated and managed effectively in order to avoid further complications such as rhabdomyolysis, which eventually lead to damage to vital organs such as kidneys. Imaging techniques such as ultrasonography, computed tomography (CT), and magnetic resonance imaging (MRI) can help in the diagnosis and evaluation of COVID-19 musculoskeletal symptoms and iatrogenic complications. In this article, we discuss the imaging characteristics and the mechanisms with which SARS-CoV-2 affects the musculoskeletal system. In particular, we review the various pathologies affecting the muscle. In extensive cohort studies, myalgia, which is characterized as muscle aches and pains, has been repeatedly observed in SARS-CoV-2 infection with a prevalence ranging from 11% to 50% [6]. Myositis and rhabdomyolysis have been mentioned in several case reports as occurring in SARS-CoV-2 infection. It can occur as both a late consequence of the infection or a presenting symptom. There have been a few isolated reports of COVID-19-affected patients with necrotizing autoimmune myositis. The mechanisms behind the involvement of the muscles in COVID-19 remain unclear. It has been shown that SARS-CoV-2 can propagate hematogenously and directly invade skeletal muscle by binding to the ACE2 receptor [6,7]. A distinct and more widely recognized notion of SARS-CoV-2 muscle involvement is immunological-mediated mechanisms, which are assumed to be after an inflammatory response with immune cell activation and massive release of cytokines. Cytokines released from the muscle due to toxicity, injury resulting from the similarity between human muscle cells and viral antigens, and deposition of immune complexes are some of the hypothesized mechanisms of immune-mediated damage to the muscle [7].

Myositis is a general term for muscle inflammation and has been linked to viral illnesses such as Hepatitis, HIV, and influenza A/B, in addition to coronavirus. A side effect of myositis is myonecrosis and myoglobinemia which is due to muscle infarction and elevated myoglobin levels in the blood due to rhabdomyolysis, respectively. Rhabdomyolysis is a potentially fatal illness that can result in intravascular coagulation, compartment syndrome, and acute renal failure [4]. Myalgia and/or weakness and increased CPK levels have both been noted in individuals affected by COVID-19, which are typical clinical signs of myositis/rhabdomyolysis [8,9]. Other illnesses causing muscle weakness or musculoskeletal symptoms such as neurological disorders like muscular dystrophies, multiple sclerosis (MS), amyotrophic lateral sclerosis (ALS), autoimmune diseases such as Graves’ disease, myasthenia gravis, thyroid conditions, and electrolyte imbalances have to be ruled out.

To establish a myopathic process and rule out analogs like motor neuron illness, electrodiagnostic investigations like electromyography (EMG) and nerve conduction studies might be helpful [10]. The gold standard for diagnosis and can be used to define locations for imaging to support the diagnosis is muscle biopsy [11]. 

The preferred imaging technique is MRI, ideally using a 1.5-T or 3.0-T magnet and including fluid-sensitive multiplanar, anatomic sequences. Muscle edema is detected as a rise in signal intensity on short tau inversion recovery sequences (STIR) or T2-weighted sequence, which is suggestive of findings of myositis [11]. Homogeneous hyperintense signal and enhancement, heterogeneous hyperintense signal, and rim enhancement are examples of illness patterns [4]. Region of necrosis or the loss of typical muscle architecture may be visible in severe illness. The "stipple sign," which consists of a region of non-enhancing muscle tissue with a surrounding rim of enhancement that contains enhancing foci within the muscle is a distinctive feature of myonecrosis [4]. On gradient echo sequences, intramuscular hemorrhage can be seen as a T1 hyperintense signal or a blooming artifact [9].

A commonly acquired condition affecting the musculoskeletal system in SARS-CoV2-affected individuals needing ICU care is critical illness myopathy. It has been also seen in association with corticosteroid use in patients on ongoing treatment for COVID-19. Corticosteroid therapy is still the key treatment and recommendation for specific critically ill patients because of its strong anti-inflammatory and anti-fibrotic effects [12]. Thus, it is one of the differential diagnoses for muscle edema noted on MRI evaluation in SARS-CoV2-affected individuals who are hospitalized. Symmetrical and widespread weakness or sudden flaccid quadriplegia are two clinical manifestations of critical illness myopathy, which is a primary myopathy. This critical illness myopathy has non-specific imaging features of widespread muscular atrophy and edema. In contrast to the rhabdomyolysis/myonecrosis associated with COVID-19, there are no signs of necrosis in critical illness myopathy. In one research of critically sick COVID-19 patients done by Cabanes Martinez et al., the degree of spontaneous activity on electrodiagnostic examinations was shown to be notably severe [13]. Patients with COVID-19 may develop dysfunction of the most principal muscle of the respiration-the diaphragm. Dysfunction may occur as a result of phrenic nerve injury, potentially as a result of the implantation of chest support devices or critical illness myopathy and/or maybe ventilator-induced diaphragm dysfunction. The SARS-CoV-2 virus could theoretically cause direct neuromuscular involvement, which could result in diaphragm dysfunction. A recent autopsy investigation done by Shi Z et al. discovered the expression of Angiotensin-converting enzyme 2 receptor in the SARS-CoV-2 virus nucleus and in the human diaphragm in a subgroup of COVID-19 patients who had diaphragm dysfunction [14]. Diaphragm dysfunction might cause respiratory problems and/or make it difficult to stop using ventilatory support in patients [15,16]. A rapid evaluation of diaphragm excursion is of utmost importance and is provided by the fluoroscopy sniff test, which is rapid and provided in real-time. Additional information is provided by ultrasound which demonstrates diaphragm muscle atrophy, examination of excursion with M mode, and the muscle thickening ratio with respiration. In the neck region, high-resolution ultrasound can also assess the phrenic nerve, which may help distinguish between neuropathic and myopathic causes of diaphragm failure. In COVID-19 patients with protracted illness, long-term muscular sequelae like sarcopenia and cachexia are noted and have been well documented [6]. Muscle loss, also known as sarcopenia or myopenia, is mostly brought on by aging; however, inactivity and poor diet can also be contributing causes. Muscle wastage is caused by a persistent condition known as cachexia. Fat infiltration and reduced muscle size are MR imaging signs of muscular atrophy, which are seen in sarcopenia and cachexia [11].

For the treatment of myositis, a variety of immunosuppressive and immunomodulatory therapeutic drugs are currently available. Glucocorticoids and immunosuppressants continue to be the first-line treatments; an early start and enough dosage can result in disease stabilization, strength recovery, and a reduction in inflammation. However, it's important not to undervalue the negative effects of immunosuppressive therapy. Early addition or escalation of therapy should be prompted by refractory cases and extra muscular symptoms, such as interstitial lung disease, heart involvement, etc. Treatment of difficult cases is dealt with by innovative treatment strategies that target particular immune pathways that show great promise. To more accurately anticipate the response to a particular treatment, it is also imperative to conduct an additional study into the pathophysiology of myositis. Together, these initiatives may, perhaps, lead to future advancements in myositis therapy [17].

## Conclusions

COVID-19 and its predictable manifestations are generally recognized and studied extensively. The unusual symptoms and signs of the SARS-CoV-2 infection need a more careful and thorough examination. In our case report, we discuss the consequence of COVID-19 in the musculoskeletal system, in particular, the muscles. MR imaging can be very helpful in determining the involvement and characteristics of the musculoskeletal disease associated with COVID-19. It can be used for both the initial diagnosis and the follow-up evaluation to estimate the progression of the illness and assess its recovery. Musculoskeletal imaging may occasionally even serve as an indication of a previously undetected SARS-CoV-2 infection. Radiologists should be acquainted with the imaging findings, incidence, and etiology of COVID-19-related musculoskeletal symptoms to interpret imaging reports and provide patients with the best intervention possible in an expeditious manner.

## References

[1] Pal M, Berhanu G, Desalegn C, Kandi V (2020). Severe acute respiratory syndrome coronavirus-2 (SARS-CoV-2): An update. Cureus.

[2] (2021). Johns Hopkins University & Medicine Coronavirus Resource Center: Homepage. https://coronavirus.jhu.edu/map.html.

[3] Revzin MV, Raza S, Warshawsky R (2020). Multisystem imaging manifestations of COVID-19. Part 1. Viral pathogenesis and pulmonary and vascular system complications. Radiographics.

[4] Revzin MV, Raza S, Srivastava NC (2020). Multisystem imaging manifestations of COVID-19. Part 2. From cardiac complications to pediatric manifestations. Radiographics.

[5] Wiersinga WJ, Rhodes A, Cheng AC, Peacock SJ, Prescott HC (2020). Pathophysiology, transmission, diagnosis, and treatment of corona- virus disease 2019 (COVID- 19): a review. JAMA.

[6] Paliwal VK, Garg RK, Gupta A, Tejan N (2020). Neuromuscular presentations in patients with COVID-19. Neurol Sci.

[7] Keyhanian K, Umeton RP, Mohit B, Davoudi V, Hajighasemi F, Ghasemi M (2020). SARS-CoV-2 and nervous system: From pathogenesis to clinical manifestation. J Neuroimmunol.

[8] Beydon M, Chevalier K, Al Tabaa O (2021). Myositis as a manifestation of SARS-CoV-2. Ann Rheum Dis.

[9] Wasserman PL, Way A, Baig S, Gopireddy DR (2021). MRI of myositis and other urgent muscle-related disorders. Emerg Radiol.

[10] Paganoni S, Amato A (2013). Electrodiagnostic evaluation of myopathies. Phys Med Rehabil Clin N Am.

[11] Smitaman E, Flores DV, Mejía Gómez C, Pathria MN (2018). MR imaging of atraumatic muscle disorders. Radiographics.

[12] Yang T, Li Z, Jiang L, Xi X (2018). Corticosteroid use and intensive care unit-acquired weakness: a systematic review and meta-analysis. Crit Care.

[13] Cabañes-Martínez L, Villadóniga M, González-Rodríguez L (2020). Neuromuscular involvement in COVID-19 critically ill patients. Clin Neurophysiol.

[14] Shi Z, de Vries HJ, Vlaar AP, van der Hoeven J, Boon RA, Heunks LM, Ottenheijm CA (2021). Diaphragm pathology in critically Ill patients with COVID-19 and postmortem findings from 3 medical centers. JAMA Intern Med.

[15] Guarracino F, Vetrugno L, Forfori F (2021). Lung, heart, vascular, and diaphragm ultrasound examination of COVID-19 patients: a comprehensive approach. J Cardiothorac Vasc Anesth.

[16] McCool FD, Tzelepis GE (2012). Dysfunction of the diaphragm. N Engl J Med.

[17] Glaubitz S, Zeng R, Schmidt J (2020). New insights into the treatment of myositis. Ther Adv Musculoskelet Dis.

